# Trials need participants but not their feedback? A scoping review of published papers on the measurement of participant experience of taking part in clinical trials

**DOI:** 10.1186/s13063-019-3444-y

**Published:** 2019-06-24

**Authors:** Claire Planner, Peter Bower, Ailsa Donnelly, K. Gillies, Katrina Turner, Bridget Young

**Affiliations:** 1 NIHR School for Primary Care Research, Centre for Primary Care and Health Services Research, University of Manchester, 5th Floor Williamson Building, Manchester, M13 9PL UK; 2 North West Hub for Trials Methodology Research, Block F Waterhouse Building, University of Liverpool, 1-5 Brownlow Street, Liverpool, L69 3GL UK; 3 Health Services Research Unit, University of Aberdeen, 3rd Floor, Health Sciences Building, Aberdeen, AB25 2ZD UK; 4 Population Health Sciences, University of Bristol, Canynge Hall, 39 Whatley Road, Bristol, BS8 2PS UK; 5 Department of Health Services Research, Institute of Population Health Sciences, University of Liverpool, Liverpool, L69 3GL UK

**Keywords:** Trial, Participation, Patient experience, Patient satisfaction, Patient-centred trials

## Abstract

**Background:**

Participant recruitment and retention are long-standing problems in clinical trials. Although there are a large number of factors impacting on recruitment and retention, some of the problems may reflect the fact that trial design and delivery is not sufficiently ‘patient-centred’ (i.e., sensitive to patient needs and preferences). Most trials collect process and outcome measures, but it is unclear whether patient experience of trial participation itself is routinely measured. We conducted a structured scoping review of studies reporting standardised assessment of patient experience of participation in a trial.

**Methods:**

A structured search of Medline, PsycINFO, Embase and CINAHL (Cumulative Index to Nursing and Allied Health Literature) and hand searching of included studies were conducted in 2016. Additional sources included policy documents, relevant websites and experts. We extracted data on trial context (type, date and location) and measure type (number of items and mode of administration), patient experience domains measured, and the results reported. We conducted a narrative synthesis.

**Results:**

We identified 22 journal articles reporting on 21 different structured measures of participant experience in trials. None of the studies used a formal definition of patient experience. Overall, patients reported relatively high levels of global satisfaction with the trial process as well as positive outcomes (such as the likelihood of future participation or recommendation of the trial to others).

**Conclusions:**

Current published evidence is sparse. Standardised assessment of patient experience of trial participation may provide opportunities for researchers to enhance trial design and delivery. This could complement other methods of enhancing the patient-centredness of trials and might improve recruitment, retention, and long-term patient engagement with trials.

**Electronic supplementary material:**

The online version of this article (10.1186/s13063-019-3444-y) contains supplementary material, which is available to authorized users.

## Background

Randomised controlled trials (RCTs) are often described as the ‘gold standard’ method for assessment of effectiveness because they offer the most rigorous way of determining cause–effect relationships [1]. Yet, despite the efforts expended to deliver trials, recruitment and retention still pose significant challenges. A review of 73 publically funded multi-centre trials in the UK—delivered through the National Institute for Health Research (NIHR) Health Technology Assessment and Medical Research Council programmes—found that only 55% recruited 100% of their target sample size within their pre-agreed timescale but that nearly 45% received an extension of some kind [2]. There is little evidence of major improvement over time [3].

Success in recruitment and retention is often considered to reflect trial design and management. However, trials are dependent on the willingness of patients to give their time and effort and to agree to randomisation and follow-up. One potential way of increasing the willingness of patients to participate is to design and conduct trials that are aligned with the ‘wants, needs and preferences’ of patients (i.e., applying the concept of patient-centred care to trials) [4].

Patient and public involvement (PPI) in research is the process of involving patients and the public in shaping the design of research and has the aim of making research responsive to the needs of potential research participants [5, 6]. There is growing evidence concerning the optimal ways to achieve effective involvement of patients in research design and emerging evidence that such work is having demonstrable benefits [7–10]. However, the focus of PPI is on patient and public *input* to trial design and delivery. There is far less focus on assessing the *output* of such work in terms of the actual experience of participants in those trials where PPI has been implemented. Comprehensive and routine measurement of patient experience in trials could provide important evidence on the effects of endeavours to make trials ‘patient-centred’.

### The concept of participant experience

There is no formal definition of patient experience in the context of trials or research. A recent review identified four aspects common to many definitions of patient experience, which are relevant for trials in the healthcare setting: (1) the sum of all interactions (2) shaped by an organisation’s culture (3) which influences patient perceptions (4) across the continuum of care [11].

Different types of patient experience have been distinguished in the literature [12]. *Preferences* are ideas about what should occur in interactions with research studies. *Reports* are objective observations of those interactions (e.g., the amount of time spent completing questionnaires as a measure of ‘research burden’). *Evaluations* are reactions to the experience of doing research, in terms of whether it was good or bad. For the purposes of this article, we were interested in reports and evaluations as measures of patient experience in trials as opposed to preferences about what should occur.

A number of different aspects of a trial may be important in terms of patient experience. These might include recruitment (information and consent), randomisation (the need for such allocation and the way it is explained and conducted), research treatment delivery, outcome measurement and follow-up, and ‘close out’ (results sharing). Patient experience of these aspects may impact on their overall satisfaction with participation and wider outcomes of participation (whether a patient would participate again or would recommend participation to friends and family).

### What are the potential benefits of patient experience measurement?

There is increasing consensus that measurement is a necessary aspect of quality improvement [13]. Traditionally, the assessment of patient experience in routine healthcare settings (outside the context of clinical trials) has been secondary with a far greater focus on outcomes. However, increasing patient involvement in healthcare decision-making has led to renewed interest in measurement of patient experience as a way of understanding the performance of healthcare systems and as a driver of quality improvement [14]. For example, in the UK, large-scale measurement of the experience of millions of primary care patients is conducted routinely and is used as a barometer of system performance and an impetus to quality improvement [15]. Although patient experience may not be accorded the same weight as health outcomes, it may be an important complement to those traditional measures.

Although traditional outcome measures will always remain the focus of trials, we argue in this paper that routine measurement of patient experience in trials could provide similar benefits to those found in routine health care settings. There is much research that has explored participants’ experience within trials but has involved qualitative research focussed on certain aspects of the trial (e.g., understanding of randomisation or informed consent) and on a subset of patients or professionals [16, 17]. Though critical to improving trial delivery by exploring the perceptions of participants and developing an understanding of the process [18], such work could be usefully complemented by structured assessments of the wider experience of the whole trial sample. Detailed qualitative evaluation is resource-intensive and may not be practical in routine trial contexts.

Most trials already undertake comprehensive measurement on patients and so have a ready platform to assess the experience of their participants. If assessment of experience were carried out on a routine basis, it might allow measurement of variation over time, between population subgroups within the trial, and could potentially allow identification of problems and challenges which may act as barriers to successful completion of current or future trials. Effective feedback loops might allow deployment of interventions to enhance participant experience and increase engagement with research—a policy goal the UK NIHR has outlined in its report *Promoting a Research Active Nation* [19].

However, at present, there is no agreed-upon standardised methodology to capture patient experience in trials, and it is unclear whether the routine measurement of patient experience is widespread. Our aim was to review the literature to identify use of standardised measures of participant experience in a trial.

Our objectives were to do the following:

- Identify studies (involving any type of participant, intervention, comparison or outcomes) using a standardised measure of patient experience of trial participation.
- Characterise the measures in terms of purpose, format and aspects of participant experience that were assessed.
- Report existing findings on patient experience within the identified studies.
- Make recommendations for future development and application of participant experience measurement.

## Method

We conducted a ‘scoping review’, which allowed us to ‘map’ this research area and provide an initial overview [20]. We reported the study according to the new guidelines for scoping reviews [21]. There was no review protocol.

### Searches

The search of databases was performed in June 2016 by the lead author CP. Searches were limited to English language articles and non-English articles with English abstracts. The reference lists of included studies, grey literature, policy documents and relevant websites were also searched, and experts in the field of clinical trials were contacted via the UK Clinical Research Collaboration (UKCRC) Registered Clinical Trials Units Network (https://www.ukcrc-ctu.org.uk/) to discuss published work and ongoing studies in this area.

### Information sources

We searched Medline, PsycINFO and CINAHL (Cumulative Index to Nursing and Allied Health Literature) from 1999 to 2016. The following search terms (text words and medical subject headings) were used: Trial* OR RCT OR treatment effectiveness evaluation AND experience OR satisfaction or patient experience OR participant experience OR attitude. We checked the reference lists of included studies for further references but did not conduct citation searches on eligible studies. There were no restrictions placed on type of trial, population or condition. Search terms were reviewed and tested for sensitivity with an information specialist. The search prioritised sensitivity over specificity.

### Study inclusion and exclusion criteria

We included studies using standardised measures which could be either patient self-report or interviewer administered. All titles and abstracts were screened by CP, and the decision to include or exclude was recorded. If multiple papers were published (e.g., reporting on different outcomes), the multiple reports were treated as a single study but all publications were referenced. Studies were managed by using Reference Manager software. We excluded measures not related to health research and measures which focus on only one aspect of a trial (e.g., recruitment and informed consent).

### Data charting and synthesis

Data were extracted from papers according to three main categories:

- Trial context (type of trial, date run, and location of sites) and population (age, gender and condition)
- Participant experience measure, which included the measure name, type (report and evaluation), administration (interviewer and self-report), and the aspects of patient experience measured
- Summary of results reported

Data were extracted and recorded by CP only. We undertook a narrative analysis of the results in line with our research objectives.

## Results

**Fig. 1:**
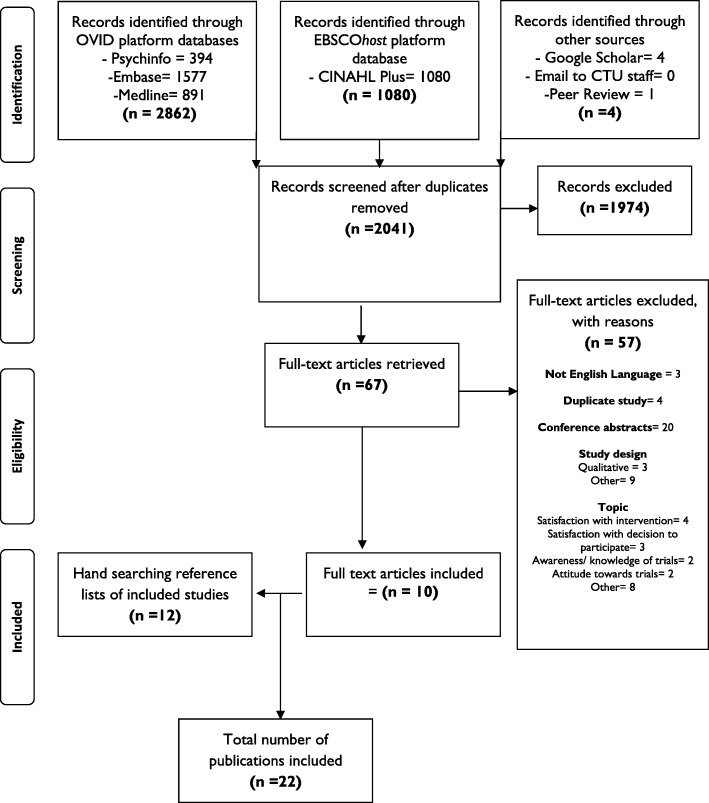
Scoping study inclusion flowchart

The search identified 2041 records, and 67 full text articles were retrieved. Fifty-seven were excluded for various reasons (the most common being a conference abstract only). Twelve additional articles were identified through searching the reference lists of those studies. We identified a total of 22 journal articles reporting on 21 different structured measures of participant experience in trials (Fig. 1). The key features of the measures are described below and summarised in Tables 1 and 2 [22–43]. (Fuller details of the included studies are presented in Additional file 1.)

**Table 1:** Summary of articles reporting on participant experience measures

Study	Geographical location(s)	Number of trials or facilities in the study	Delivery and administration	Items	Participants surveyed (response rate)	Domains
Almeida *et al*. (2007) [22]	Portugal	1 facility	Self-report In person Before discharge from the study	14	136 (100%)	i) Motivations for taking part ii) Perception of informed consent iii) Perception of study participation
Aman *et al*. (1995) [23]	Not reported	2 trials	Self-report Postal 4 weeks after enrolment	6	63 (63.5%)	i) Staff characteristics ii) Study operations iii) Specific features of trial iv) Incentives and reinforcements
Bahati *et al*. (2010) [24]	East Africa	3 facilities	Interviewer Not reported Study end	Not reported	Not reported (98%)	i) Satisfaction with service received ii) Satisfaction with major components of the research study
Bertoli et al. (2007) [25]	Argentina	3 trials	Self-report Postal Not reported	34	114 (94.6%)	i) Socio-demographics ii) Overall trial satisfaction iii) Quality of information given by the Investigator iv) Participants’ self-perception of knowledge about randomised controlled trials (RCTs) v) Objective evaluation of participants’ knowledge about RCTs
Bevan *et al*. (1993) [26]	UK	1 trial	Interviewer In person Not reported	Not reported	199 (99%)	i) Reasons for participation or declining participation ii) Aspects of trials disliked
Cain *et al*. (2005) [27]	UK and Republic of Ireland		Self-report Postal Study end	23	44 (82%)	i) Information provided ii) Reasons for taking part iii) Best and worst aspects of participation iv) Willingness to participate in the future
Dias *et al*. (2005) [28]	Not reported	1 trial	Self-report Postal 3.5 year follow-up	19	469 (88%)	i) Staff characteristics ii) Study operations iii) Specific features of trial iv) Incentives and reinforcements
Fearn *et al*. (2010) [29]	UK	1 trial	Self-report Postal Study end	Not reported	910 (59%)	i) Motivations for taking part ii) Health professional involvement iii) Randomisation iv) Filling in questionnaires v) Experience of participation
Friesen *et al*. (2016) [30]	USA	1 trial	Self-report In person At final clinic appointment	47	180 (98%)	i) Attitudes towards trials ii) Working with study team iii) Perception of risk benefit iv) General satisfaction
Van Gelderen, *et al*. (1993) [31]	The Netherlands	10 trials	Self-report Combination of postal, taken home and at end of trial Study end	12	153 (94%)	i) Reason for participation ii) Information received iii) Most unpleasant aspects iv) Most pleasant aspects v) Experience of participation
Hassar *et al*. (1976) [32]	USA	1 trial	Interviewer Not reported Study end	12	1503 (80%)	i) Reasons for investigators taking part ii) Participant impression of clinical studies iii) Medical practice in general
Henzlova *et al*. (1994) [33]	USA and Canada	1 trial	Self-report Not reported Before close out visit	10	4751 (74%)	i) Primary motivation for participation ii) Satisfaction with participation and perceived outcome iii) Effect on health-conscious behaviour iv) Negative experiences
Kost *et al*. (2013) [34, 35]	USA	29 facilities	Self-report Not reported Study end	76	18,890 (29% of participants surveyed)	i) Informed consent ii) Trust iii) Coordination of care iv) Information, education and communication v) Respect for participant preferences
Luzurier *et al*. (2015) [36]	France	1 trial	Self-report Not reported Not reported	Not reported	210 (% not reported)	i) Satisfaction (with overall welcome and protocol management) ii) Motivation for taking part iii) Participation outlook
Martin *et al*. (2011) [37]	USA	3 trials	Self-report Postal Not reported	47	75 (89%)	i) Understanding of participation ii) Reasons for participating iii) General experience iv) Overall satisfaction v) Willingness to participate in a future study including a placebo-controlled trial
McAdam *et al*. (2002) [38]	Not reported	1 trial	Not reported Not reported Not reported	7	21 (81%)	i) Information provided ii) Staff iii) Research processes iv) Research outcomes v) Willingness to participate in the future
Mattson *et al*. (1985) [39]	USA	1 trial	Self-report/Interview Postal Before final follow-up	5	1503 (80%)	i) Perceived benefits ii) Perceived beneficiaries iii) Perceived disadvantages iv) Perceived effects of medication v) Participation in a future trial
Renfroe *et al*. (2002) [40]	USA and Canada	1 trial	Self-report Postal Study end	7	664 (71%)	i) Would participate again ii) Main reasons for participation iii) Study benefits iv) Study problems v) Quality of care.
Schron *et al*. (1997) [41]	USA	1 trial	Self-report Postal Final follow-up (4.5 years after enrolment)	10	4281 (82%)	i) Reason for participating ii) Satisfaction with participating
Tangrea *et al*. (1992) [42]	USA	1 trial	Self-report Postal Study end	7	891 (97%)	i) Benefits ii) Most unpleasant aspects iii) Importance iv) Satisfaction v) Physical well-being vi) Willingness to take active treatment if shown to be effective vii) Participation in future trials
Verheggen *et al*. (1998) [43]	The Netherlands	26 trials	Personal interview (at start of the trial) Telephone interview (3 months later)	Not reported	Personal interview = 172 (93%); Telephone interview = 172 (78.5%); control group (only participating in telephone interview) = 34 (100%)	i) Medical technical aspects ii) Interpersonal iii) Organisational aspects

**Table 2:**
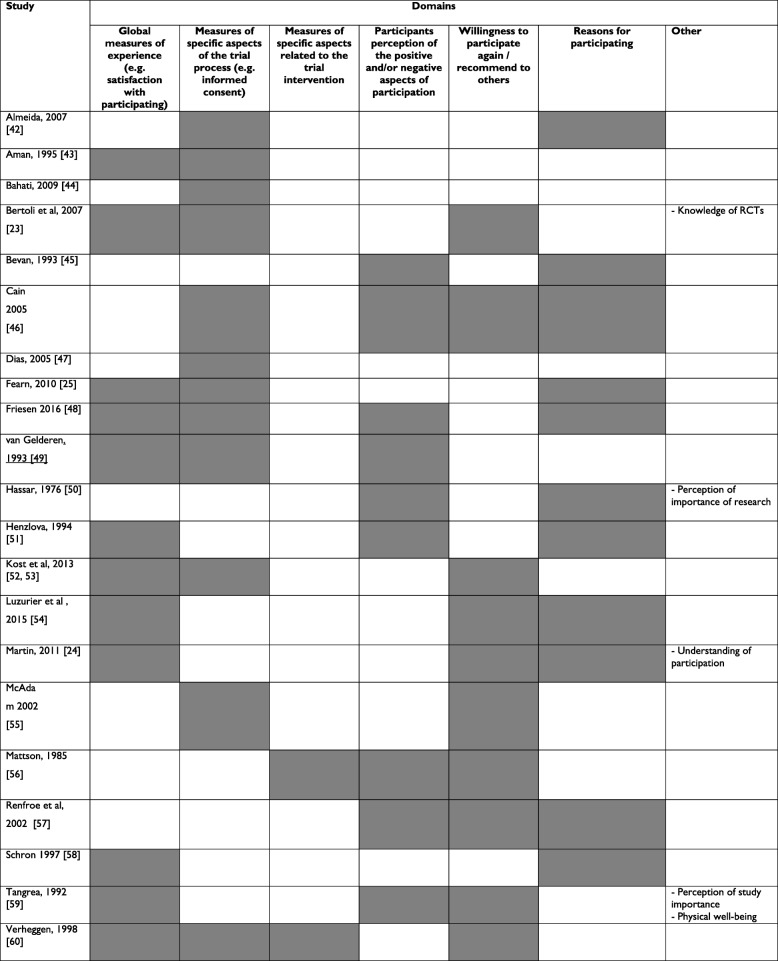
Summary of domains measured (shaded boxes represent domains measured)

### Trial context

Of the measures identified, the majority assessed experience on single trials (*n* = 13). The majority (*n* = 12) were published since 2000 and were conducted in the United States (*n* = 7), United States and Canada (*n* = 2), or Europe (n = 7). The measures assessed participant experience in varied clinical contexts, including cancer care, dentistry, arthritis and emergency medicine.

### Types of measures within the trials

All of the measures focussed on *evaluations* (reactions to the experience of doing research, in terms of whether it was good or bad) rather than *reports* (objective observations of interactions; e.g., the amount of time spent completing questionnaires).

### Defining participant experience and measure development

None of the articles offered a formal definition of participant experience, and only one reported a psychometric analysis which included validity and reliability testing.

### Format, mode of delivery and time points

Where reported, the administration of the measure was by self-report (*n* = 15) or interview (*n* = 4) or a combination (n = 1) and measures were sent to participants by post (*n* = 10) or used in person (*n* = 3) or a combination (n = 1).

Of the 16 articles that reported the number of items included in the measure, the mean was 21 (range of 6–76). None of the articles reported the completion time for either self-report or interviewer administered measures. Of those that reported the administration time point, the majority were administered at the end of the trial (*n* = 14), one study measured 4 weeks after enrolment, and only one study required participants to complete a measure on more than one occasion.

### Participants

Participant characteristics were reported in 19 of the articles, and there was considerable variation in the range of characteristics reported. Three articles reported the percentage of people who withdrew from the trial but completed the measure, which ranged from 3.9% to 8.6%.

### Domains assessed

As summarised in Table 2, content of the measures varied, but common aspects included global measures of experience (such as satisfaction with overall experience), measures of specific aspects of relevance to all trials (such as informed consent), measures of aspects relevant to trial interventions (such as treatment side effects), and outcomes (such as willingness to participate again or the likelihood of recommending a trial to family and friends).

Some studies included additional measures which were not related to the specific experience of the trial but might be important drivers of that experience (such as motivation for taking part and expectations around experience). None of the studies asked participants for their feedback on the participant experience measure itself.

### Results of the patient experience measures

Given the variability in the trials and study populations, comparison of the results must be made with caution. Response rates to the patient experience measure varied but were broadly in line with what might be expected from the usual rates achieved in trial outcome measures. Response rates ranged from 29% to 100%. Of those studies reporting response rates, 15 (75%) out of 20 reported rates over 80%, which is usually seen as a marker of quality [44].

Overall, participants reported high levels of global satisfaction with the process. When asked, a majority of participants suggested that they would participate in trials in the future, and 11 (85%) out of 13 studies reported that outcome. Nevertheless, some trials did report less positive experience from a significant minority of participants. For example, one in five participants did not rate information and informed consent highly in one study [25], whereas in another study, nearly half felt that participation interfered with their family routine [37]. In another study, only 52% reported willingness to participate in further research in another [29]. There were insufficient studies to permit any sensible assessment of factors across studies related to participant experience.

## Discussion

### Summary of results

Our search of the literature found a relatively small number of eligible studies. Measuring participant experience does not appear to be common in the published literature, making it difficult to quantify key aspects of experience, such as levels of satisfaction or dissatisfaction with trial processes, or to explore patient or trial characteristics associated with satisfaction. However, there may be a significant grey literature that our search failed to uncover.

From the limited data presented, it would seem that generally respondents express high levels of satisfaction and are positive about further participation and recommendations to others. Although this is an important finding, there is a significant potential for publication bias (as results critical of a trial may be less likely to be published), and it is possible that patients who are likely to take part in trials may have experienced higher-quality care more generally. Even in the context of the broadly positive results, some of the trials did report less positive experience from a significant minority of participants.

It is not clear how much focus should be placed on the experience of patients participating in trials compared with those preferences and experiences of people who do not participate in trials. Obviously, in many trial contexts, the latter far outnumber the former. Of course, it is possible that the experience of patients in trials will provide insights which can translate to better proportions of patients being recruited in the first place. However, the drivers of participation and good experience may be different.

### Limitations of the studies

Studies had a number of limitations, including the lack of a formal definition of patient experience and the use of measures without detailed data about their development or psychometric characteristics. However, these limitations are to be expected in a developing research area. Response rates to the patient experience measures were generally acceptable, but clearly there is the potential for bias if patients with particular experiences are less likely to return measures, along with participants who withdraw or are eligible but decide not to participate. As with trials more generally, research could usefully explore ways to maximise return rates [45, 46].

### Strengths and limitations of the review

We have characterised our study as a scoping review, as we did not conduct a formal quality appraisal. The search was designed to provide a reasonable balance between sensitivity and the resources available to the review. Assessment of papers and data extraction were conducted by a single reviewer. We do not feel that these limitations are critical given our focus on scoping the current evidence on the use of standardised questions and the very limited evidence reported. There is no standardised system for quality assessment of the types of studies included in the scoping review, which could have been assessed as surveys (through criteria such as response rates and data completeness) and as measurement studies (in terms of psychometric criteria such as reliability and validity). This would have required a potentially complex assessment which may not have been proportionate given the aims of the scoping review.

Our search for the scoping review was relatively circumscribed in order to keep the yield of the search manageable. The search was conducted in mid-2016, and resource limitations meant that we have been unable to update the search. It is possible that new studies have been published, although we would not expect major changes in the evidence base or the conclusions of our review. As with any search, the focus was on published work and we may have missed unpublished work carried out by trial teams or research units. There may be a wider literature in the commercial sector, where there is much current interest in concepts such as ‘patient-centricity’ as applied to trials. We did not contact industry experts as part of our scoping work. Our discussions with local trial teams do not suggest that measurement of patient experience is widespread, although some research contexts (such as dedicated research facilities) may be better able to conduct this sort of work, and the Clinical Research Network in the UK has begun systematic work on patient experience [47]. If there is ‘hidden literature’ about what happens in trials (in internal reports or in the tacit knowledge of investigators), it would be important to understand how that could be better reported and used.

The author team has tried to ensure a patient perspective on the issues raised in this paper. Workshops exploring the concept of a ‘patient-centred’ trial were run alongside this scoping review (http://research.bmh.manchester.ac.uk/patientcentredtrials/resources/) and patients were involved in those workshops alongside a range of professional stakeholders. Author AD is a patient representative who has had a long-standing involvement in this project, and our future funded work in this area will include extensive PPI. Nevertheless, it is important to be aware of the tension between the patient perspective on trial participation and the interests of professional stakeholders, which are often (though not exclusively) focussed on recruitment and retention. We expect that, in many cases, the goal of improving patient experience will be aligned with the outcomes of improved recruitment and retention, but it is important to be aware that there may be cases in which there is tension between them (e.g., where retention may be enhanced by proactive follow-up, which some patients may find burdensome).

### Implications

From the literature identified by our review, it would appear that information about participant experience is not systematically reported on individual studies, trial units, centres or research facilities. As well as providing feedback for research staff on individual trials, standardised assessment could be aggregated to allow assessment of participant experience across multiple trials across a trials unit or across a funder’s portfolio. This might allow identification of broader trends which need higher-level intervention.

Before adoption of standardised measurement of participant experience, there are many issues that need consideration. We outline some recommendations for future research in this area, in terms of both the practical issues about *how* data are collected and wider issues concerning the meaning and interpretation of the data.

### Practical issues concerning the collection of patient experience data

- *What are the core dimensions of a ‘patient-centred’ trial that should be included in a participant experience measure?* Table 2 highlights variation in what aspects of patient experience are measured in different studies and suggests that global experience, specific aspects of the trial (such as informed consent) and positive and negative aspects of participation are most likely to be measured. It is not clear which aspects are most important to patients or other stakeholders (such as trial teams and funders) or how evaluations of the different aspects are associated, as they may reflect a global assessment. Effective priority-setting methods such as those used in previous assessments of patient priorities around trials may be useful in this regard [48]. It will be important to identify the generic questions of relevance to all trials and others that may be important for particular trials or in particular contexts. A modular approach to measurement (with generic and trial-specific measures) may be optimal.
- *What are the optimum format and delivery mode of patient experience measures?* Further work is required to understand the optimum way in which to collect experience data. All of the studies found in our review measured evaluations rather than reports, although it is unclear why that is the case. Most studies evaluated experience at the end of the study, which allows a more comprehensive assessment of the entire experience of participation but raises issues concerning the ability of participants to recall earlier aspects of the trial.
- *What is the correct balance between quantitative and qualitative approaches to measuring patient experience?* Clearly, there is a significant qualitative literature on patient experience in trials [16, 49] and it will be important to explore the optimal methods by which they can complement each other to take advantage of their relative strengths.
- *How can developments in technology facilitate measurement?* Developments in technology may improve the collection of patient experience data in the future. For example, digital recording of patient narratives might be analysed by using text mining to allow efficient capture of data that is richer than standardised measures.
- *Should measurement of patient experience be independent of the trial team?* Independence may better avoid bias and the perception of pressure but that may not be feasible in the context of limited resources available to trial teams. The impact of such independence on assessments could be assessed by using the Study-Within-A-Trial design [50].

### Issues in the interpretation of patient experience data

- It is important to understand what influences patient experience and how much of the variation in experience is due to context and trial type, patient characteristics, or aspects of the trial. There is an ongoing debate as to whether adjusting for such factors is a fairer way of assessing performance or whether such adjustment removes the imperative to improve care [51].
- In terms of factors related to the trial itself, it will be important to determine how much of the variation in patient experience is due to specific processes (how patients are approached, how consent is gained, and preferences considered) [52] compared with the general interpersonal and communication skills of staff [53]. Effective ‘closure’ in trials (thanking patients for participation and providing results) may be as important as their experience in the trial itself [54].
- Another methodological issue of interest is whether patients can distinguish between their experience of the interventions within a trial and their experience of the other trial procedures. Acceptability of interventions will often be assessed in pragmatic trials as part of a comprehensive assessment of the value of the intervention. Some aspects of patient experience may be beyond the control of the trial team (such as the result of their randomised allocation and the outcomes patients achieve from treatment).
- It will be important to explore the relative importance placed on the measurement of patient experience in different types of trials. For example, some trials have little active patient participation or even awareness of participation (such as cluster trials without individual consent), where a focus on patient experience may be less relevant.
- It will also be important to consider the costs and other disadvantages of a focus on patient experience in trials. There may be potential unintended consequences of measures of patient experience (such as causing trial teams to focus on aspects of experience that are easily measurable compared with more complex issues). Work in this area will also have to be aware of the wider literature on the concept of ‘satisfaction’ and its measurement [55–57].

Although measurement of participant experience may be necessary for quality improvement, it is unlikely to be sufficient. It will be important to assess what other facilitators and resources need to be in place to ensure that results lead to improvement and that trial teams ensure a ‘virtuous circle’ between measurement, feedback, and the design and delivery of trials. We will be exploring how data can be used for quality improvement in our ongoing funded work, drawing on published examples of the use of feedback in other contexts [58]. The wider literature in audit and feedback would suggest that positive impacts are most likely when baseline performance is poor and when the feedback comes from a colleague (which in this context might be other trialists rather than others sharing a particular professional background). Developing a ‘virtuous circle’ would require regular feedback, using multiple formats, with clear targets and a plan for remedial action [59]. Adoption of appropriate theory may have an important role to play [60].

### Recommendations for describing participant experience measurement

We identified some deficiencies in the reporting of the use of patient experience measures in our scoping review. We recommend reporting on the following:

- Whether the measure was used to assess experience in one trial or across a trial portfolio.
- Trial context (trial phase, condition under investigation, and core features of intervention)
- Location where the trial or trials were conducted (country and facility where applicable)
- Development detail (e.g., how the items were selected, estimated completion times, and whether the measure has been subject to reliability and validity testing)
- Number of participants invited to complete the measure and response rate
- Percentage of participants who complete the measure who withdrew from the trial (failure to adhere to the protocol or provide routine follow-up data)
- Participant characteristics, including demographics (and other characteristics which may be relevant to specific trial/facility)
- Delivery mode (postal, face-to-face interview, telephone interview, and online) and administration time points
- Any incentives or tokens of thanks given to participants for completing the measure
- Number of items and response options (if the measure is not published as part of the article)
- Details for how the measure can be sourced (and languages available).

## Conclusions

The regular and standardised assessment of participant experience of trials could provide useful feedback for trial teams, complement other methods of assessing patient experience, and assist in the development of patient-centred trials. At present, there is little evidence that measurement is conducted routinely. We outline key questions in this area to promote research around this issue.

## Additional file

Additional file 1: Structured measures of participant experience reported in peer-reviewed journal articles. (DOCX 67 kb)

## Data Availability

All data generated or analysed during this study are included in this published article and its supplementary information files.

## Abbreviations

NIHR: National Institute for Health Research
PPI: Patient and public involvement
RCT: Randomised controlled trial
