# Supplementary material for: Trials need participants but not their feedback? A scoping review of published papers on the measurement of participant experience of taking part in clinical trials
Source: Trials. 2019 Jun 24;20:381. doi: 10.1186/s13063-019-3444-y (PMC6591815; doi:10.1186/s13063-019-3444-y)
Supplement: Supplementary file 1 — Structured measures of participant experience reported in peer-reviewed journal articles. (DOCX 67 kb) [file 13063_2019_3444_MOESM1_ESM.docx]

**Additional file 1 : Structured measures of participant experience reported in peer reviewed journal articles**

| **Study** | **Study details & aim** | **Definition of patient experience; measure; format** | **Population** | **Domains measured and results** | **Other results** |
| --- | --- | --- | --- | --- | --- |
| Almeida, 2007 [22] | **Trial or Clinical Facility:** Participants in trials taking place within one facility- the Human Pharmacology Unit  **Trial phase:** Phase I  **Trials conducted:** Not reported (participants surveyed Jan 2006- July 2006)  **Location:** Portugal  **Aim:** characterize motivations and attitudes of health volunteers and perceptions of informed consent and participation | **Definition:** Not reported  **Name of measure:** Not reported  **Validated:** Not reported  **Format:** self-report (also completed the NEO Personality Inventory)  **Mode of delivery:** At the research facility  **Time-points:** Before discharge from the study  **Items:** 14  **Response options:** Structured  **Language:** Not reported (English version of questions reported in results section) **Available:** Not reported  **Incentives:** Yes receive financial incentive (amount not specified) | **Research participants surveyed**: 136  **Response rate:** 100%  **Trial withdrawals completing Q:** Not reported  **Respondent characteristics:**  Age: mean = 26.9 (±5.5)  Ethnicity: Caucasian = 98.5%; other = 1.5%  Occupation: Student = 51.9%, employed = 42.2%, unemployed = 5.9%  Net income: <25% = 23.5%, 25%-50% = 28.7%, 51%-100% = 28.7%, 101%-150% = 12.2%, >150% = 7.0%  Level of education: Master’s degree = 2.9%, licensed= 21.5%, Bachelor’s degree = 6.7%, 12 years= 60%, 9 years = 7.4%, 6 years = 1.5%  Habituation: parents/family home= 40.7%, participants own home = 26.7%, rental house/ flat = 17.8%, rental room = 9.6%, university facility = 5.2%  Civil status: single = 72.6%, married/living together = 21.5%, divorced = 5.9% | **Domain descriptors** i) motivations for taking part ii) perception of informed consent ii) perception of study participation  **Results**  **Motivations for taking part**  - Financial reward most important single motivation - extremely important / ‘5’ (58.4%), important / ‘4’ (30.1%); Contribution to scientific progress – extremely important (19.1%), important (27.9%); Free medical check-up – very important (20.6%), important (30.9%); Curiosity – very important (8.1%), important (27.2%)  - Would take part without financial reward – no (38%), maybe (52.9%), yes (8.8%)  - Perception of financial reward – good (33.8%), reasonable (62.5%), bad (3.7%)  - Heard about opportunity – poster (5.1%), flyer (7.4%), HPU staff (16.2%), Ex-participant (69.1%), someone who never participated (13.2%), other (2.2%).  - Before participating was someone else’s opinion- no (11.8%), yes (88.2%), family/partner (53.3%), friend (40%), physician (25%), unspecified (20.8%), other (4.2%)  - How did family/ close friends react- almost all said yes (20.3%), some said yes, some said no (46.8%), almost all said no (32.6%), all said no (1.9%)  - If recommended no, reasons- risk (753.7%), guinea pig role (40%), missing work/school (8.7%), away from home (7.8%), other (1.7%)  **Perception of informed consent**  - Information provided before the study- more than enough (44.4%), enough (54.1%), not enough (1.5%)  - Inconvenience/discomfort in relation to information provided - lower than informed (34.6%), similar to informed (58.8%), higher than informed (6.6%)  - Feelings about the possibility of a serious complication- never feared that (81.6%), occasionally (16.2%), significant period of time (2.2%)  **Perception of study participation**  - Confidence in skills of clinical staff- always sure (95.6%), occasional doubts (4.4%)  - Participate again – (0.7%), maybe (14%), yes (85.3%)  - Would recommend to friend/relative – no (0.7%), maybe (10.3%), yes (89%) | **Moderators:** No significant correlations between age, gender, principal occupation and the motivators for participating  **Mediators:** Participants with lower income and lower education considered financial reward more important than participants on a lower income.  - Personality traits correlated with decision to ask someone else’s opinion (higher in neuroticism, self-consciousness, impulsiveness and vulnerability and lower in order, achievement striving, self-discipline, self-conscientiousness). Those who asked for clinician opinion were higher in achievement striving and gregariousness.  - Personality traits correlated with the manner in which information provided. Those who considered information provided as ‘more than enough’ relatively higher in warmth, positive emotions, feelings, tender-mindedness and competence.  - Personality traits correlated with perception of the risk of participation. Those who perceived greater risk showed a higher anxiety trait. |
| Aman, 1994 [23] | **Trial or Clinical Facility:** x2 trials in children (one with Risperidone and one with Pondimin)  **Trial phase:** Not reported  **Trials conducted:** Not reported  **Location:** Not reported  **Aim:** To assess social importance and personal benefit attached to involvement in drug research | **Definition:** Not reported  **Name of measure:** Not reported  **Validated** Not reported  **Format:** Self-report  **Mode of delivery:** Postal  **Time-points:** 4 weeks post involvement  **Items:** 6  **Response options:** Closed  **Language:** English  **Available:** From journal article  **Incentives:** None reported | **Research participants surveyed**: 63  **Response rate:** 63.5%  **Trial withdrawals completing Q:** Not reported  **Respondent characteristics:** Not reported | **Domain descriptors:** i) Acceptability of study procedures ii) Satisfaction with study outcomes  **Results across both trials**  **Acceptability of study procedures**  - 100% felt assessments were important (97% for learning tests)  - 100% felt that study personnel were friendly and responsive to concerns  - 98% felt visits were convenient  - 83% felt q’s were satisfactorily answered regarding role of medication for their child  **Satisfaction with study outcomes**  - 88% satisfied with individual conclusions reached and information provided  - 88% would join the study again | **Moderators:** None reported  **Mediators:** None reported |
| Bahati, 2009 [24] | **Trial or Clinical Facility:** x3 research centres  **Trial phase:** Cycles 1 and 3  **Trials conducted:** August 2006- July 2007  **Location:** East Africa  **Aim:** To adapt a QI model for health services for use in prevention research settings | **Definition:** Not reported  **Name of measure:** Not reported  **Validated:** Not reported  **Format:** Exit interview by someone outside of study team  **Mode of delivery:** Not reported  **Time-points:** Study end  **Items:** Not reported  **Response options:** Structured and qualitative  **Language:** Not reported  **Available:** Not reported  **Incentives:** None reported | **Research participants surveyed**: Not reported  **Response rate:** 98% of those approached in cycle I and 100% in cycle 2 (52% of total enrolled)  **Trial withdrawals completing Q:** Not reported  **Respondent characteristics:** 50.5% male | **Domain descriptors:** i) Satisfaction with service received ii) Satisfaction with major components of the research study  **Results reported cycle 1:**  **Satisfaction with service received and major components of the research study**  - Attitude of community mobilizers very good (94%), welcomed by the receptionist (97%), waited less than 30 minutes to see counselor (62%), informed consent revisited (90%), counselor discussed benefits of HIV test disclosure (97%), family planning discussed with counsellor (66%), attitude of Doctor or trial physician very good (86%), were welcomed, listened to and treated respectfully (100%), understood advice given about health (100%), waited less than 15 minutes to receive drug (or to see pharmacist) (70%), received a very good service (87%), waited less than 30 minutes to see Dr/ trial physician (99%)  **Results reported cycle 2 following QI:**  - Attitude of community mobilizers very good (80%), welcomed by the receptionist (100%), waited less than 30 minutes to see counselor (97%), informed consent revisited (96%), counselor discussed benefits of HIV test disclosure (98%), family planning discussed with counsellor (95%), attitude of Doctor or trial physician very good (94%), were welcomed, listened to and treated respectfully (98%), understood advice given about health (98%), waited less than 15 minutes to receive drug (or to see pharmacist) (100%), received a very good service (94%), waited less than 30 minutes to see Dr/ trial physician (100%) | **Moderators:** None reported  **Mediators:** None reported |
| Bertoli et al, 2007 [25] | **Trial or Clinical Facility:** x3 RCTs (osteoarthritis and rheumatoid arthritis)  **Trial phase**: Not reported  **Trials conducted:** March 2002 – August 2003  **Location:** Argentina  **Aim:** To assess participant’s knowledge of informed consent and explore whether knowledge level is related to clinical trial satisfaction | **Definition:** No definition provided  **Name of measure:** Not reported  **Validated:** No  **Format:** Self-report survey (illiterate responders could complete with assistance)  **Mode of delivery:** Postal  **Time-points:** Completed within one year of consenting for RCT.  **Items:** 34  **Response options:** 31 items had structured response items (multiple choice) and 3 were open-ended  **Language**: Spanish  **Available:** Not reported  **Incentives:** no incentives for trial participation (travel expenses reimbursed)  or completing survey | **Research participants surveyed**: 114  **Response rate:** 94.6%  **Trial withdrawals completing Q**: 8.6%  **Respondent characteristics:** Mean age= 56.3 (SD 11.8); 87.4% female; 59% rheumatoid arthritis 41% osteoarthritis; median duration of patient-physician relationship= 5 years (IQR 2-9.25); 1.9% responders were illiterate, 48.6 % attended elementary school, 28.6% completed high school, 19% achieved higher degrees; 66% participating for first time | **Domains descriptors**  i) Socio-demographics ii) Overall trial satisfaction iii) Quality of info given by the Investigator iv) Participants' self-perception of knowledge about RCTs vi) Objective evaluation of participants' knowledge about RCTs  **Results reported:**  **Overall trial satisfaction**  - 91.4% said main reason for participating was expectation of getting better and/or receiving free treatment (46.7%), 41.9% said altruism (collaborating with physician), and/ or altruism (collaborating with Science)- 33.3%  - 99% were satisfied overall  **-** 96.2% would participate in a future research study  - 93.2% felt better since enrolling  - 6%% reported better than usual medical care, 35% said it was the same  - 56.7% said relationship with rheumatologist improved, 43.3% said it did not change  - 86.4% reported no inconvenience while participating, 5.8% said they were inconvenienced because the treatment was not effective, 5.8 were inconvenienced because they did not tolerate the medication, 1.9% because they spent money.  **Quality of info given by the Investigator**  - 81% rated the quality of information given during informed consent as high quality; 15.2% intermediate; 3.8% low.  - 82.9% Felt informed consent form was very easy to understand  **Participants' self-perception of knowledge about RCTs**  - 51% self-reported having a good level of knowledge of RCTs  **Objective evaluation of participants' knowledge about RCTs** (NB unclear how assessed)  - 14.3% qualified as high following objective evaluation. | **Moderators:**  - Age negatively associated with higher levels of knowledge  - No association between education and knowledge  **Mediators:**  -No significant correlation between knowledge and satisfaction  **Other:**  - 88.6 informed consent taken by patient’s physician  - 83.5% Participants fully read informed consent form; 11.7% partially read it; 9% didn’t remember  - Informed consent form required a 10^th^ grade education to understand |
| Bevan, 1993 [26] | **Trial or Clinical Facility:** Group A -66 previous or current participants; Group B- 12 decliners ; Group C- 119 not invited  **Trial phase:** Not reported  **Date conducted:** Not reported  **Location:** Glasgow, UK  **Aim:** Explore attitudes to participation in therapeutic drug trials | **Definition:** Not reported  **Name of measure:** Not reported  **Validated:** Not reported  **Format:** Interviewer administered  **Mode of delivery:** Not reported  **Time-points:** Not reported  **Items:** Not reported  **Response options:** Not reported  **Language:** English  **Available:** Not reported  **Incentives:** None reported | **Research participants surveyed:** 199  **Response rate:** 99%  **Trial withdrawals completing Q:** Not **Respondent characteristics**:  Group A- Mean age 56.8±9.7; female (41%); Abbreviated Mental Test score >9 (98%); socio-economic group= i and II (9%), III and IV (36%), V and VI (37%) | **Domain descriptors** i) Reasons for participation/ declining participation  ii) Aspects of trials disliked  **Results**  **Reasons for participation:**  To help others (62%), to improve own treatment (39%), because asked by  Dr (38%), to get info about disease (5%), out of gratitude to hospital (5%), persuaded by family (2%), to pass time (2%), curiosity (2%)  **Aspects of trials disliked:**  - Nothing to dislike (54%)  - Fear of side effects (4%), venepuncture (9%), wearing a 24hr blood pressure monitor (7%), time commitment (4%)  **Other:**  - 38% would discuss participation with friends and family  - 38% were given written information to retain | **Moderators:** None reported  **Mediators:** None reported |
| Cain 2005 [27] | **Trial or Clinical Facility:**  **Trial phase:** phase III  **Trials conducted:** Not reported  **Location:** UK and Republic of Ireland  **Aim:** To examine the attitudes and experiences of participants | **Definition:** Not reported  **Name of measure:** Not reported  **Validated:** Not reported  **Format:** Self report  **Mode of delivery:** Postal with pre-paid return to central collection point (not trial centres)  **Time-points:** Trial end  **Items:** 23  **Response options:** Combination of smiley faces (scale of 5), categorical responses and numerical, free text options for some q’s (adapted from Wong and Baker)  **Language:** English  **Available:** Not reported  **Incentives:** None reported | **Research participants surveyed**: 44 (aged 6-17)  **Response rate:** 82%  **Trial withdrawals completing Q:** Not reported  **Respondent characteristics:** 6-11 years old (17%); 12-14 (25%); 15-17 (17%). | **Domain descriptors** i) Information provided ii) Reasons for taking part iii) Best and worst aspects of participation iv) Willingness to participate in the future  **Results reported:**  **Information provided**  - 94% felt they had been given enough information about the trial  - 75% gave colourful supplementary patient information leaflet a positive score compared to 59% for the main patient information leaflet.  **Reasons for taking part**  - 30% said main reason for participation was to control blood sugar; 21% thought it would be interesting, 19% to help other people with diabetes, 9% because mum/dad thought it would be a good idea, 2% because friend was doing it  **Best aspects of participation**  - 56% learned more about their condition, 13% trying new insulin/eating after injecting, 3% more attention on me, helping others 13%, 5% food /trial aids, 10% time off school  **Worst aspects of participation**  21% blood sampling, 10% getting up early, 10% none, 10% diary completion, 13% night time blood glucose reading*% more injections, 5% fasting blood glucose readings  **Willingness to participate in the future**  - 81% would participate in another trial  - 72% positive about trial diary (56% wanted more colour or pictures and 17% wanted clearer instructions), 92% positive about glucose meter, 86% happy with more frequent visits | **Moderators:**  - all age groups said ‘learning about diabetes’  Was best part of being in a trial  - all age groups said ‘blood sampling’ was  Worst part about being in a trial. The 15-17  Year olds also rated ‘night time glucose  Readings’ and ‘more injections’ equally as  Bad.  **Mediators:** None reported |
| Dias, 2005 [28] | **Trial or Clinical Facility:** Correction of myopia evaluation trial  **Trial phase:** Not reported  **Trials conducted:** 1997-2003  **Location:** Not reported  **Aim:** Evaluate reasons for COMET’s high retention | **Definition:** Not reported  **Name of measure:** Not reported  **Validated:** Not reported  **Format:** Self-complete at visit or at home if unable to complete at visit  **Mode of delivery:** Posted in advance and returned at next visit  **Time-points:** At 3.5 year follow-up (2001-2002)  **Items:** 19  **Response options:** 4 point scale indicating level of preference for each item  **Language:** English  **Available:** Not reported  **Incentives:** None reported | **Research participants surveyed**: 469 families  **Response rate:** 88%  **Trial withdrawals completing Q:** Not reported  **Respondent characteristics:** Mean age 9.3; 47% male; 25.6 African American, 47.2% white, 14.1 hispanic | **Domain descriptors** i) Staff characteristics ii) Study operations iii) Specific features of trial iv) Incentives and reinforcements  **Results**  **Staff characteristics**  - 100% liked a lot or somewhat study staff  **Study operations**  **-** 100% liked a lot or somewhat appointment reminders and convenience of appointments, 75% liked a lot or somewhat location/ access of centres  **Specific features of trial**  - 100% liked a lot the free eyeglasses  - 100% liked a lot or somewhat repair of glasses, association with college of optometry, and being part of a nationwide study  - 97% liked a lot or somewhat the completeness of the eye exam  - 80% liked a lot or somewhat the COMET commitment  - 71% liked a lot or somewhat the length of the study  - 89% liked a lot or somewhat the selection of frames  - 17% liked a lot or somewhat eye drops  **Incentives and reinforcements**  - 97% liked a lot or somewhat the thank you materials  - 98% liked a lot or somewhat the newsletters | **Moderators:** None reported  **Mediators:** None reported |
| Fearn, 2010 [29] | **Trial or Clinical Facility:** Mineral and Vitamin Intervention Study (MAVIS trial)  **Trial phase:** Not reported  **Trials conducted:** Not reported  **Location:** Scotland, UK  **Aim:** Factors influencing older people to take part and remain in an RCT | **Definition:** Not reported  **Name of measure:** Not reported  **Validated:** Not reported  **Format:** Self-report  **Mode of delivery:** Postal  **Time-points:** After the end of the trial  **Items:** Not reported  **Response options:** Open and closed  **Language:** English  **Available:** Not reported  **Incentives:** Not reported | **Research participants surveyed**: 910  **Response rate:** 59%  **Trial withdrawals completing Q:** Withdrawals not surveyed  **Respondent characteristics:**  Age: mean 72 years  Gender: female (48%); male (52%)  Trial arm: Active (49%), placebo (50%) | **Domain descriptors** i) motivations for taking part ii) health professional involvement iii) randomization iv) filling in questionnaires vi) experience of participation  **Results**  **Motivations for taking part**  **-** Helping other people - very important (86%); a little important (22%); not at all important (2%)  - Helping the research team- very important (86%) ; a little important (11%); not at all important (1%)  -Reducing risk of illness- very important (37%); a little important (41%); not at all important (17%)  **Health professional involvement**  - Being asked by a Dr - very important (42%); a little important (27%) ; not at all important (23%)  - Being asked by a nurse- very important (28%); a little important (34%) ; not at all important (31%)  **Randomization**  - Having treatment decided by chance - very important (21%); a little important (30%); not at all (42%)  - Having the possibility of taking a dummy tablet - very important (11%); a little important (24%); not at all (60%)  - having to take tablets – very important (21%%); a little important (25%); not at all (50%)  - knowing what is in the tablets - very important (26%); a little important (27%); not at all (42%)  **Filling in questionnaires**  -having to fill in questionnaires or diaries- very important (24%); a little important (29%) ; not at all (41%)  **Experience of participation (open ended questions)**  Positive aspects of trial participation reported: good communication and friendly personal contact, trial organisation and conduct of trial staff, simplicity and ‘straightforward’ nature of the involvement  Negative aspects of trial participation reported: infection diaries and questionnaires, uncertainty of placebo and taking pill.  - 52% willing to participate in future research. Most frequently reported reason for not wishing to take part =perception of age and poor health status | **Moderators:** None reported  **Mediators:** None reported |
| Friesen 2016 [30] | **Trial or Clinical Facility:** Dental clinical trial  **Trial phase:** Not reported  **Trial conducted:** Not reported  **Location:** Midwestern Academic Dental Centre, USA  **Aim:** examine reasons for participation in trials, to inquire where they learned about the trial, and to gain feedback regarding their experiences of participating | **Definition:** Not reported  **Name of measure:** Qualtrics survey  **Validated:** Not reported  **Format:** Self-report  **Mode of delivery:** At appointment; completed in a private area  **Time-points:** Final clinical evaluation appointment  **Items:** 47  **Response options:** 5 point likert scale  **Language:** English  **Available:** Not reported  **Incentives:** None reported | **Research participants surveyed**: 180  **Response rate:** 98%  **Trial withdrawals completing Q:** Not reported  **Respondent characteristics:** Women (67.1%); White (59.8%), African American (25%), Hispanic (7.9%), other (6.1%);Age 30-44 (37.2%), 45-59 (37.2%); Higher college degree (54.4%); Yearly income >$50,000 (43.7%) | **Domain descriptors** i) Attitudes towards trials ii) Working with study team iii) Perception of risk benefit iv) General satisfaction  **Results reported for subscale domains (strong agreement = 5):**  **Relatively strong agreement on the following:**  - Need for dental research (4.7; SD 0.8)  - Satisfaction with study consent / communication processes (mean- 4.3; SD 0.8)  - Personal reasons for participating (mean 4.1; SD 0.7)  - Sense of autonomy in choosing to participate (4.6; SD 0.7)  - Study satisfaction (mean- 4.6; SD 0.5)  **General disagreement for the following:**  - Negative attitude towards participating (mean- 1.6; SD 0.7)  - Negative perception of care received during study (mean 1.7; SD 0.6)  **Less clear perception:**  - Fate/god role in outcomes (mean- 3.1; SD 1.2)  - Participating for financial reasons (mean 2.7; SD 1.1) | **Moderators:**  - African Americans more likely to rate Fate /God as important to health and participation in research compared to white Americans  - African Americans more likely to rate that their social norms do not support being involved in research compared to white and other Americans  .45-59 year olds less likely to attribute financial reasons to for participation compared to other groups.  **Mediators:**  -Those with less education more likely to rate Fate/God as important to health and participation, lower levels of autonomy for participation compared to college graduates |
| Van Gelderen, 1993 [31] | **Trial or Clinical Facility:** 10 experiments  **Trial phase:** Not reported  **Trials conducted:** 1989- 1991  **Location:** Netherlands  **Aim:** To explore motives of healthy volunteers for participating, evaluate information provided and experience of participation | **Definition:** Not reported  **Name of measure:** Not reported  **Validated:** Not reported  **Format:** Self-report  **Mode of delivery:**  1 experiment mailed q within 1 month; 1 experiment q was taken home; 8 experiments q provided at end of experiment  **Time-points:** At end of each experiment  **Items:** 12  **Response options:** Mostly multiple choice with option for additional comments, 1 open question  **Language:** English  **Available:** From journal  **Incentives:** Financial compensation for participating in trial | **Research participants surveyed**: 153  **Response rate:** 94% overall (100% in 7 experiments, 73%, 81% and 92% in the other 3 experiments)  **Trial withdrawals: completing Q:** Not reported  **Respondent characteristics:** Age 18-30 (76%), >61 (17%); Male (67%); Education- Higher Vocational (15%), University (67%); 67% pursue sports, 25% sometimes; 14% smoke, 22% sometimes, 64% no; 46% drink alcohol, 46% sometimes, 8% do not; Health is very important to me (61%), important (20%), Most important (19%) | **Domain descriptors:** i) Reason for participation ii) Information received iii) Most unpleasant aspects iv) Most pleasant aspects vi) Experience of participation  **Results (data pooled across 10 experiments)**  **Reason for participation**  For money (65%), other reasons (22%), other items (14%)  **Information received**  - Sufficient (85%), insufficient (13%), Too much (1%)  -Information corresponded to study (77%), did not (17%), No idea beforehand (5%)  **Most unpleasant aspects**  Food (13%), blood sampling (19%), collection of urine (11%), staying in ward (20%), other (55%)  **Most pleasant aspects**  Food (17%), blood sampling (7%), collection urine (5%), staying in ward (23%), other 42%), personal contact with staff and other volunteers (30%)  **Experience of participation**  - Participants who would participate again with knowledge they have now (89%), no (2%), maybe (8%)  -Participants who would participate again (80%), would not (1%), maybe (18%) | **Moderators:**  - Correlation between reason for participating and age- 96% of younger volunteers aged 18-30 participated due to financial compensation; most of volunteers who cited ‘due to a check up’ were 60+ (75%); for benefit of others >61 (83%)  **Mediators:**  - Volunteers who felt financial compensation unreasonable were positive about participating in a future trial  **Other:**  Lower response rate to questionnaires taken home or posted to home |
| Hassar, 1976 [32] | **Trial or Clinical Facility:** RCT of an anti-inflammatory drug  **Trial phase:** Not reported  **Trial conducted:** Not reported  **Location:** USA  **Aim:** To explore reasons for the study, views of clinical studies and medical practice in general | **Definition:** Not reported  **Name of measure:** Reasons for volunteering and attitudes about drug studies in humans  **Validated:** Not reported  **Format:** Interview with researcher?  **Mode of delivery:** Not reported  **Time-points:** Study end  **Items:** 12  **Response options:** 6 multiple choice, 6 open-ended  **Language:** English  **Available:** From journal  **Incentives:** None reported | **Research participants surveyed**: 1503 (representing a sample of 50% of living BHAT participants)  **Response rate:** 80% across the two questionnaires-  questionnaire A (n= 594) questionnaire B (n= 608); 21% of inactive participants and 82% of active participants  **Trial withdrawals completing Q:** 3.9% described as inactive  **Respondent characteristics:** Mean age 54 [30-69) | **Domain descriptors:**  i) Reasons for investigators taking part ii) Participant impression of clinical studies and iii) Medical practice in general  **Results reported:**  **Reasons for investigators taking part- open ended responses presented numerically**  To help patients arthritis (16/17), to help other patients (15/17), to help the University, to help the drug company (3/17)  **Participant impression of clinical studies- open ended responses presented numerically**  Things that impressed most – thoroughness and dedication of investigators (9/17), physical examinations and blood tests (4/17), Interest shown by participants (2/17)  Things that were a ‘turn-off’- not getting better (2/17), differences in examination techniques between physicians (1/17), getting an ulcer (1/17), impression that physician was not convinced of benefit of drug (1/17)  **Views on drug studies**  Some are necessary (9/17), all are necessary (8/17); Drug studies are of some scientific value (4/17), great scientific value (13/17)  **Views on medical practice**  Most Doctors are concerned with patient wellbeing (17/17 agree), Doctors you have known are concerned with patient wellbeing (16/17 agree), Doctors make too much money (1/17 agree), must be a better way to finance medical care (9/17 agree), drug company research is necessary (16/17 agree), drug companies make too large a profit (3/17 agree). | **Moderators:** None reported  **Mediators:** None reported |
| Henzlova, 1994 [33] | **Trial or Clinical Facility:** Long-term heart failure trial  **Trial phase:** Not reported  **Trial conducted:** Not reported  **Location:** USA, Canada.  **Aim:** To explore participants’ attitudes and perceptions at the end of a long term trial | **Definition:** Not reported  **Name of measure:** SOLVD Close-out Questionnaire  **Validated:** Not reported  **Format:** Self-report  **Mode of delivery:** Not reported  **Time-points:** Immediately before the close out clinic visit  **Items:** 10  **Response options:** Closed-responses  **Language:** English  **Available:** Not reported  **Incentives:** None reported | **Research participants surveyed**: 4751  **Response rate:** 74% Sub-groups - 33% participated in treatment trial and 67% participated in prevention trial  **Trial withdrawals completing Q:** Not reported  **Respondent characteristics:** 87% male, 8% reported previous participation in trial. | **Domain descriptors:** i) Primary motivation for participation ii) Satisfaction with participation and perceived outcome iii) Effect on health-conscious behaviour iv) Negative experiences  **Results reported:**  **Primary motivation for participation**  31% Advice of primary physician, 20% to contribute to medical science, 12% to help others, 15% to live longer, 10% to feel better, 1.4% for free medical care and free medication  **Satisfaction with participation and perceived outcome**  97% satisfied with participation with expectations fulfilled, 96% would recommend to a friend, 87% would participate in another trial; 62% perceived emotional improvement, 56% physical improvement  **Effect on health-conscious behavior**  15% Stopped smoking, 15% stopped drinking and 38% changed diet.  **Negative experiences**  21% Travel to and from clinics, 16% difficulty in finding parking, 7% frequent staff changes | **Moderators:** Females stressed possible personal benefit over altruism; females more likely to perceive trial as having a positive emotional impact; men more likely to enroll in a future trial  Change of diet reported more frequently by females.  **Mediators:**  **-**Incidence of bypass surgery and/or angioplasty and smoking history slightly higher in responders Vs non-responders  - Treatment trial participants stressed possible personal benefit over altruism  - Prevention trial participants more likely to perceive trial as having a positive emotional impact  -Patients who had previously participated more likely to stop smoking  -Treatment trial participants more likely to abstain from alcohol  **Other:**  -Average completion time reported as 10 minutes  - Participation not anonymous |
| Kost et al, 2013 [34,35] | **Trial or Clinical Facility:** 15 Clinical Research Centres (supported by the NIH), 13 Clinical and Translational Science Award Sites, 1 General Clinical Research Centre, NIH Clinical Research Centre  **Trial type/ phase:** Not reported  **Trials conducted:** Not reported  **Location:** US  **Aim:** To develop and validate a standardised survey | **Definition:** No definition, but example of PE provided:  *“...whether the*  *informed consent process properly and completely prepared them for a research study”*  **Name of measure:** Research  Participant Perception Survey (DVS)  **Validated:** Validation study  **Format:** Self-report  **Mode of delivery:** Not reported  **Time-points:** After study end (precise time-point NR)  **Items:** 76  **Response options:**  Combination of scale and structured response format (dichotomous yes/no; multiple option)  **Language**: English, Spanish  **Available:** through journal article  **Incentives:** None reported | **Research participants surveyed**: 18, 890  **Response rate:** 29% of those delivered, range 18%-74% (some sites restricted population which survey was sent to)  **Trial withdrawals completing Q** : Not reported  **Respondent characteristics:** 85% white; 59% female (of Centers recording sex); 37% healthy volunteers; 54% completed 4 years+ of college education or higher; 51% had participated in only 1 study; 36% participated in studies that were months long in duration, 31% were years long in duration; 19% rated health status as excellent, 36% as very good, 29% as good, 13% as fair and 2% as poor. | **Domains descriptors**  i) informed consent ii) trust iii) coordination of care iv) information, education and communication vi) respect for participant preferences  **Results reported:**  **Overall experience**  - 73% rated overall experience very highly  **Would recommend to friends and family**  -66% would definitely recommend to friends and family; 31% would probably do so  **Pressure from staff to join**  - 94% Never felt pressure to join; 6% sometimes, usually or always felt pressure to join (combined)  **Consent form explained**  - 81% felt the risks of joining were always included in the consent form; 19% said usually, sometimes or never (combined)  **Study details included in the consent form**  - 80% said study details were included in the consent form; 20% said no  **Time to think about decision to participate before signing the consent form**  - 79% said yes they had enough time; 21% said no  **Consent form written in a way that could be understood**  - 78% said they always understood the consent form; 22% said usually, sometimes or never (combined)  **Consent form was preparation for what to expect**  - 67% said the consent form prepared them for what to expect; 25% said mostly and 8% somewhat or not at all  Coordination of care  **Study results**  - 23% received a summary of results; of those that did not 85% would like to have; 72% rated having a summary of results as ‘very important’  **Trust**  - 86% trusted the research team completely; 99% felt they had been treated with courtesy and respect; 79% felt they were treated as valued partners; 93% were listened to carefully by Investigators and 95% by research coordinators; | **Moderators:** white participants had a higher level of trust- 88% giving the highest rating; 78%-82% of non-whites gave the highest rating.  **Mediators:**  - More likely to rate overall experiences very highly if trusted the PI and nurses, were treated with respect, listened to, given understandable answers to q’s; and could meet with the PI as much as wanted.  - Patient experience similar at sites with high and low response rates.  **Other:**  - Readability of questionnaire score 6.8 |
| Luzurier [36] | **Trial or Clinical Facility:** RCT efficacy of different telephone techniques to assist untrained volunteers in performing cardiac massage for vital emergency  **Trial phase:** Not reported  **Trial conducted:** Sept- Nov 2013  **Location:** France  **Aim:** To explore factors associated with healthy volunteer (HV) motivation and satisfaction with participation in RCTs (without financial compensation) | **Definition:** No definition provided  **Name of measure:** Not reported  **Validated:** Not reported  **Format:** Self-report  **Mode of delivery:** Not reported  **Time-points:** Not reported  **Items:** Not reported  **Response options:** Structured response format (5-item Likert scale); multiple choice (several answers possible)  **Language:** French  **Available:** Not reported  **Incentives:** Stated that no financial compensation | **Research participants surveyed:** 210 healthy volunteers  **Response rate:** Not reported  **Trial withdrawals completing Q:** Not reported  **Respondent characteristics:** mean age= 43.5 years (SD 12.4); male: female ratio= 0.5; healthy volunteers without professional activity (12.5%), students (15.5%), blue-collar (5%); employees (40.5%), mid-level professionals (13.5%), managers/intellectual professionals (13.5%); 46.2% had previously participated in a study. | **Domains descriptors**  i) Satisfaction (with overall welcome and protocol management) ii) Motivation for taking part iii) Participation outlook  **Results reported:**  **Satisfaction**  - 49% very satisfied with overall welcome  - 40% very satisfied with protocol management  **Motivation for taking part**  - 86.5% were motivated to take part to support medical research, 85.7% to update knowledge on emergency first aid.  **Participation outlook**  - 98.5% would participate in a new research study  - 99% would recommend participation to relatives | **Moderators:**  - Women significantly more likely to take part to support medical research  - Older respondents significantly more likely to take part due to interest in medical research results; younger respondents due to curiosity  - Overall satisfaction with welcome higher for older respondents (46-60 years) and those in management / intellectual professional jobs  - Satisfaction with protocol management higher for women and older respondents (46-60 years)  **Mediators:**  - Respondents registered on a research database were significantly more likely to take part to support medical research |
| Martin, 2011 [37] | **Trial or Clinical Facility:** Facility (3 trials)  **Trial phase:** Phase III  **Trials conducted:** Not reported  **Location:** USA  **Aim:** Assess experience of parents of children with neurofibromatosis (NF1) participating in trials | **Definition:** Not reported  **Name of measure:** Not reported  **Validated:** Reliability testing not conducted due to heterogeneity of items  **Format:** Self-report on paper or online  **Mode of delivery:** Postal  **Time-points:** Not reported  **Items:** 47  **Response options:** Multiple choice, likert (typically 4 point) and open-ended **Language:**  **Available:** Not reported  **Incentives:** None reported | **Research participants surveyed**: 75 families (89% currently or previously taking part, 11% evaluated to participate but did not participate)  **Response rate:** 85%; (84% returned by paper, 16% returned online)  **Trial withdrawals completing Q:** Not reported  **Respondent characteristics:** 86% mothers, 14% fathers; 59% of children male; 25% <6 years old, 33% 6-10, 42% 11-24 | **Domain descriptors** i) Understanding of participation ii) Reasons for participating iii) General experience iv) Overall satisfaction vi) Willingness to participate in a future study including a placebo-controlled trial  **Results reported:**  **Understanding of participation**  - 86% had a thorough understanding of purpose of trial, 92% medical procedures involved, 86% possible side effects, 86% time required for participation, 96% right to withdrawal  - 100% of those taking part in a placebo controlled trial (PCT) said they understood reason a lot or somewhat, 52% had no issue taking part in a PCT  **Reasons for participating**  77% to shrink child’s tumour, 72% to access medical care otherwise unavailable, 72% to help others, 56% increasing knowledge of NF1, 28% meeting other families living with NF1  **General experience**  -47% felt participation interfered with their family routine ‘somewhat’ or ‘a lot’  - 75% felt it impacted family relationships in a positive way ‘some’ or ‘a lot’  - 78% did not have sig. financial difficulty attending the centre  - 77% no notable difficulties with child taking time of school  - 47% felt co-workers/ supervisors not understanding about time off work required  - 92% amount of contact with staff appropriate  - 84% felt very comfortable calling research staff with q’s  - 80% said staff members were very easy to reach  - 72% felt it helped child ‘some’ or ‘a lot’  - 28% said worst part was taking time off work, 25% travelling to centre, 25% child having to miss school  **Overall satisfaction**  77% said ‘opportunity to help others’ best part about taking part, 69% access to medical care expensive outside the facility, 58% learned more about NF1  **Willingness to participate in a future study including a placebo-controlled trial**  - 84% would definitely participate in a future study, 9% probably  - 40% definitely and 26% probably would participate in a placebo-controlled trial. | **Moderators:** None reported  **Mediators:**  - Participating in a PCT or treatment study had no impact on satisfaction  **-** higher satisfaction significantly correlated with fewer transportation issues, fewer financial difficulties, fewer school problems for the child resulting from participation |
| Mattson, 1985 [39] | **Trial or Clinical Facility:** RCT of Beta-Blockers for Heart Attack (BHAT) and RCT of Aspirin for Myocardial Infarction (AMIS) study  **Trial phase:** Not reported  **Trial conducted:** Not reported  **Location:** USA  **Aim:** To explore perceptions of advantages and disadvantages of trial participation | **Definition:** Not reported  **Name of measure:** Not reported  **Validated:** Not reported  **Format:** AMIS – qualitative interviews; BHAT- questionnaire A, questionnaire B,  **Mode of delivery:** Postal  **Time-points:** Before final follow-up before allocation unblinded  **Items:** 5  **Response options:** Closed ended and 5 point Likert scale; response categories developed in response to AMIS findings.  **Language:** English  **Available:** Not reported  **Incentives:** None reported | **Research participants surveyed**: 1503 (representing a sample of 50% of living BHAT participants)  **Response rate:** 80% across the two questionnaires-  questionnaire A (n= 594) questionnaire B (n= 608); 21% of inactive participants and 82% of active participants  **Trial withdrawals completing Q:** 3.9% described as inactive  **Respondent characteristics:** Mean age 54 [30-69) | **Domain descriptors:** i) Perceived Benefits ii) Perceived beneficieries iii) Perceived disadvantages iv) Perceived effects of medication iv) Participation in a future trial  **Results reported:**  **Perceived Benefits**  -72% treatment and information from heart specialists, 76% increased knowledge of physical condition, 65% free physical exams, 60% free lab results, 63% peace of mind, 54% personal awareness/education, 37% physical improvement, 35% free medication, 17% interaction with heart patients.  **Perceived beneficiaries**  Self (participants) 59%, scientists 82%, BHAT staff 80%, other heart patients 73%, family members 54%, friends 30%  **Perceived disadvantages**  Transportation (11%), time-consuming clinic visits (10%), scheduling inconvenience (5%), changes in clinic staff (4%), location of clinic (4%), rushed visits (2%)  **Perceived effects of medication**  43% felt much better or somewhat better overall as a result of the medication, 46% felt the same, 11% felt worse; 51% not sure that medicine would prevent another heart attack, 38% slightly or fairly sure it would, 11% very or extremely sure  **Participation in a future trial**  78% said yes of (which 51% definitely yes, 27% probably, 16% not sure and 3% would not) | **Moderators:** Greater proportion of non-responders were nonwhite (26% vs 9%), smokers (65% vs 53%), female (20% vs 15%), slightly higher blood cholesterol and systolic blood pressure.  **Mediators:** Participation seen as important to general and cardiac health despite uncertainty of whether medicine would prevent a future attack.  **Other:** Perceived benefits categories similar in BHAT and AMIS; altruistic motivations high in both, perceived disadvantages low in both |
| McAdam 2002 [38] | **Trial or Clinical Facility:** Trial of atypical neuroleptic risperidone in treating severe aberrant behavior in people with developmental disabilities  **Trial phase:** Not reported  **Date conducted:** Not reported  **Location:** Not reported  **Aim:** Assess caregivers satisfaction with participation of their child in a trial | **Definition:** Not reported  **Name of measure:** Consumer Satisfaction Survey  **Validated:** Not reported  **Format:** Not reported  **Mode of delivery:** Not reported  **Time-points:** Not reported  **Items:** 7  **Response options:** Closed responses  **Language:** English  **Available:** From journal article  **Incentives:** None reported | **Research participants surveyed**: 21 primary caregivers (e.g., parents, guardians) of persons with ‘mental retardation’  **Response rate:** 81%  **Trial withdrawals completing Q:** Not reported  **Respondent characteristics:** Not reported | **Domains** i) Information provided ii) Staff ii) Research processes iv) Research outcomes vi) Willingness to participate in the future  **Results**  **Information provided and conclusions reached**  -53% satisfied with conclusions reached & information provided (47% satisfactory)  -70.6% said usefulness of medication was definitely addressed, 17.6% somewhat, 5.9% not as much as hoped, 5.9% unsatisfactory  **Staff**  94.1% described staff as very friendly and responsive, 5.9% somewhat  **Research processes**  -100% said assessments were obtained from researchers were important  -81.3% described appointments as definitely convenient, 12.5% satisfactory, 6.3% inconvenient  **Willingness to participate in the future**  82.4% would join the study again, 17.6% uncertain | **Moderators:** None reported  **Mediators:** None reported |
| Renfroe et al, 2002 [40] | **Trial or Clinical Facility:** Multi-centre RCT (Antiarrhythmic drugs -AAD versus implantable defibrillators ICD).  **Trial phase**: Not reported  **Trial conducted:** 1993-1997  **Location:** US and Canada  **Aim:** To collect information about study related issues and to test different survey distribution methods | **Definition:** No definition provided  **Name of measure:** The AVID study Participant Questionnaire  **Validated:** Not reported  **Format:** Self-report (mailed to home)  **Mode of delivery:**  Survey mailings were stratified by four factors  i) overnight express mail Vs regular mail; ii) certificate of appreciation (yes vs no); iii) timing of administration ("early" vs. "late"); IV) cover letter signed by a physician vs coordinator  **Time-points:** Completed at study end. Time points defined as ‘early’ (2-3 weeks after last follow-up) or ‘late’ (1-4 months)  **Items:** 7  **Response options:** 6 structured response items (multiple choice) and 1 open-ended question  **Language:** English  **Available: From j**ournal article  **Incentives:** Certificate of appreciation of cover letter signed by a physician vs coordinator | **Research participants surveyed:** 664  **Response rate:** 71%  **Trial withdrawals completing Q:** Not reported  **Trial participant characteristics:** Average age= 65; 79% male; 81% coronary artery disease, 67% history of myocardial infarction; 49% history of congestive heart failure. | **Domains descriptors**  i) Would participate again ii) Main reasons for participation iii) Study benefits  iv) Study problems vi) Quality of care.  **Results reported:**  **Would participate again**  - 85% would participate again; 10% maybe; 6% no  **Main reasons for participation**  - 43% took part because physician recommended; 15% for closer monitoring; 14% to live longer  **Study benefits**  - 37% cited increased medical knowledge as main benefit; 23% improved health;21% frequent physician contact  **Study problems**  - 20% cited side effects as the main problem with treatment  - 29% cited transportation as main non-treatment related problem; 27% excessive waiting in clinics; 26% parking and 15% clinical personnel changes.  **Quality of care**  Not reported | **Moderators:**  - Respondents more likely to be older, Caucasian, high school graduates and live with others (multivariate analysis also  **Mediators:**  - Non responders more likely to have diabetes, unexplained syncope, dilated cardiomyopathy, Medicare/ Medicaid or Health Maintenance Organization insurance  - More likely to return if survey was delivered by overnight express mail; with no certificate of appreciation enclosed; administered close to study end. A signed thank you letter did not affect response rates.  - Treatment type (AAD vs ICD) differed in terms of reported benefits, problems with side effects  - No significant difference for non-treatment related problems  - Participants reporting non-treatment related problems less likely to participate again |
| Schron 1997 [41] | **Trial or Clinical Facility:** Systolic Hypertension in Elderly Program (SHEP)  **Trial phase:** Not reported  **Trials conducted:** Not reported  **Location:** USA (multi-centre trial)  **Aim:** Identify reasons for participation and satisfaction with participation | **Definition:** Not reported  **Name of measure:** Not reported  **Validated:** Not reported  **Format:** Self-report  **Mode of delivery:** Postal  **Time-points:** Last follow-up visit (4.5 years from study start)  **Items:** 10  **Response options:** Structured  **Language:** English  **Available:** Reported in journal article  **Incentives:** Not reported | **Research participants surveyed**: 4281  **Response rate:** 82% (higher in participants who were younger, white, more educated who did not have an MI or stroke during participation)  **Trial withdrawals completing Q:** Not reported  **Respondent characteristics:**  Age: 60-69 (43%), 79-79 (45%), 80+ (12%)  Ethnicity: black (14%), white and other (86%)  Gender: Male (42%), female (58%)  Education: <12 years (35%), ≥12 years (65%) | **Domain descriptors:** i) reason for participating ii) satisfaction with participating  **Results**  **Reasons for participating**  % important / very important  - To improve health of others (96%), to contribute to science (96%), to improve own health (93%), free medical care (65%), someone to talk with (35%), some place to go (19%)  **Satisfaction with participating**  % agree  - Staff were friendly (99.8%), staff provided good care (99.4%). SHEP will provide important information (99.2%), didn’t mind taking SHEP medication (94.2%), transportation was a problem (15.6%), too many visits (7.8%)  - 98% would recommend SHEP or a similar programme to a good friend | **Moderators:** Free medical care more important to participants who were older, black or with less years in education  Having someone to talk to more important to participants who were female, older, black, or with less years in education  **Mediators:** Not reported |
| Tangrea, 1992 [42] | **Trial or Clinical Facility:** Long term cancer chemoprevention trial  **Trial phase:** Not reported  **Trial conducted:** February 1984 – June 1987  **Location:** USA  **Aim:** Explore perceived benefits, disadvantages, importance, and satisfaction with trial participation | **Definition:** No definition provided  **Name of measure:** Not reported  **Validated:** Not reported  **Format:** Self-report  **Mode of delivery:** Postal  **Time-points:** Study end (3 years post enrolment). Completed questionnaires returned within 1 month of receipt.  **Items:** 7  **Response options:** closed ended (requiring choice of one response); other option available for q’s on perceived benefits and disadvantages  **Language:** English  **Available:** From journal article  **Incentives:** None reported | **Research participants surveyed**: 891  **Response rate:** 97% (only 793 patients completing questionnaire before notification of treatment group included in analysis)  **Trial withdrawals completing Q:** Not reported  **Respondent characteristics:** mean age 61.8 years; 77% male; 62% college or higher education; 60% reported side effects; 50% 7+ skin biopsies. | **Domain descriptors:**  i) Benefits ii) Most unpleasant aspects iii) Importance iv) Satisfaction vi) Physical well-being vii) Willingness to take active treatment if shown to be effective x) Participation in future trials  **Results reported:**  **Benefits**  Careful medical follow-up received (43%), being part of a research effort (24%), potential for reducing skin cancer (17%), overall improvement in patient care (6%), other (2%)  **Most unpleasant aspects**  Time taken to attend clinic (32%), side effects from study medication (20%), Number of blood samples (11%), chance of taking treatment or placebo (8%), taking capsules every day (8%), additional medical procedures undergone (5%), number of questions asked at each visit (2%), other (4%)  **Importance**  Study participation is very/extremely important to general health (62%), fairly important (22%), not/slightly important (14%)  **Satisfaction**  Study participation is very/extremely important to skin cancer condition (88%), fairly important (6%), not/slightly important (3%)  **Physical well-being**  As a result of participation, feel definitely/probably better physically (52%), no different (43%), probably/definitely not better (3%)  **Willingness to take active treatment if shown to be effective**  Definitely/ probably willing to take active treatment (78%), depends (14%), probably/definitely not (6%)  **Participation in future trials**  Would definitely/probably take part in a future trial (79%), depends (16%), probably/definitely not (4%) | **Moderators:**  -Careful medical follow-up cited more frequently by responders with a college or higher education  -Being part of a research effort cited more frequently by women and responders educated to high school level or less  - Older responders and those with a high school education or less more frequently cited that participation was very/extremely important to general health; younger responders in higher education group more frequently cited it as fairly important and responders with college of higher education cited it as not or slightly important  - Larger proportion of men, those in placebo group and those not experiencing side effects expressed willingness to take study medication if shown to be effective  -A larger proportion of men indicated that would be willing to take part in a future trial  **Mediators:**  -Placebo responders cited ‘amount of time to attend clinic’ as most unpleasant aspect of participation; treatment responders cited ‘medication side effects’ |
| Verheggen, 1998 [43] | **Trial or Clinical Facility:** 26 clinical trials at the University Hospital Maastricht  **Trial phase:** Not reported  **Trials conducted:** Not reported  **Location:** Maastricht, The Netherlands  **Aim:** Assess satisfaction of patients participating in trials | **Definition:** patient satisfaction was subdivided into medical-technical, interpersonal and organizational aspects of trial participation  **Name of measure:** Not reported  **Validated:** Not reported  **Format:** Personal interview and subsequent telephone interview  **Mode of delivery:** 1 hour Personal interview (within 1 month of agreeing to participate); telephone interview  **Time-points:** i) personal interview at the start of the trial ii) telephone interview conducted 3 months after personal interview  **Items:** Not reported  **Response options:** five-point Likert scale  **Language:** English  **Available:** From the authors  **Incentives:** Not reported | **Research participants surveyed**: Personal interview = 172; Telephone interview = 172; control group (only participating in telephone interview)= 34  **Response rate:** Personal interview= 93% ; Telephone interview = 78.5%; control group = 100%  **Trial withdrawals completing Q:** Not reported  **Respondent characteristics:** Not reported | **Domain descriptors** i) medical technical aspects ii) interpersonal iii) organizational aspects  **Results**  **Medical technical aspects**  **Satisfaction with own personal benefit and benefit to future patients**  **-** 58.5% experienced some personal benefit by participating  - 22.7% experienced an improvement in health as a result of trial treatment  - 61% satisfied that trial might be of benefit to future patients  **Satisfaction with medical treatment**  - 98.5% were satisfied or very satisfied with medical treatment provided  **Satisfaction with learning about health condition**  -59.2% obtained more insight into one’s health; 69.2% were more disappointed about obtaining insights than they had expected to be.  **Satisfaction with extra check-ups**  - 68.9% felt comforted by extra health check-ups; 78.8% felt less satisfied with this than expected.  **Interpersonal aspects**  **Satisfaction with information disclosure**  -93% satisfied with information disclosure; 6.7% felt the clinician should have given more explanation  **Satisfaction with trial clinician as a person**  - 94.1% had a positive attitude to trial clinician  - 97.8% had trust in personal approach of trial clinician  - 100% saw clinician as competent and conscientious  - 98.2% felt clinician had high degree of control over treatment risks  **Organizational aspects**  **Satisfaction with the way discomforts were experienced and effort had to be made to participate in the trial**  - 3.7% (54.2% had some dissatisfaction with time spent on trial because it was more than expected)  **Satisfaction in relation to compliance with trial participation**  **-** 12% considered dropping out on one or more occasions during the trial.  -91.9% felt that trial treatment should be finished when agreeing to participate; 6.7% did not agree with this; 1.4% did not answer  -3.7 asked for another medical treatment; 93.3% did not; 3% did not remember  **Participation in future**  - 88.9% definitely yes, 8.9% definitely no, 2.2% undecided | **Moderators:** None reported.  **Mediators:**  -Patients expecting risk of trial to be high were less satisfied with personal benefit they claimed to have from it.  -Patients expecting to be treated as a special kind of patient were highly satisfied with personal benefit they claimed to have from it.  -The more risks perceived the more unpleasant the medical treatment was judged to be  - Patients with little trust in medical experiments were relatively dissatisfied with obtaining insights into their health condition  - Patients with a negative attitude towards clinical research were relatively dissatisfied with obtaining insights into their health condition  - Patients with low level of trust in physicians in general felt dissatisfied with extra check-ups  - Patients that perceived their health severity to be low felt more dissatisfied with the extra check-ups  - Patients with more trust in medical experiments or more interest in medical affairs more satisfied with information disclosed.  - Patients who worried a lot about their health were much more satisfied with information discloses  - Patients expecting to be treated as a special kind of patient were had a more positive view of the trial clinician  - Patients in diagnostic trials were more satisfied with gaining insight into health condition, medical treatment and personal benefit they believe they have received and more comforted by extra check-ups than patients in therapeutic trials  - Patients in invasive trials were more satisfied with gaining insight compared to patients in non-invasive trials  - Patients in medication trials perceived more improvement in their health compared to people in therapeutic intervention trials  - Patients in RCTs found to have obtained more insight into health condition, felt more secure because of health check-ups, perceived more benefit from participating and were more satisfied with medical treatment compared to patients in non-randomised studies  - Patients in therapeutic experiments learned more about the state of their health, more satisfied with the state of their health and perceived their chances of receiving better medical treatment as higher compared to patients in non-therapeutic trials |
